# Analysis of the Influence of the Numerical Relation in Handball During an Organized Attack, Specifically the Tactical Behavior of the Center Back

**DOI:** 10.3389/fpsyg.2019.02451

**Published:** 2019-11-12

**Authors:** João Nunes Prudente, Américo Ramos Cardoso, Ana Jose Rodrigues, Duarte Filipe Sousa

**Affiliations:** ^1^Department of Physical Education and Sports, Faculty of Social Sciences, Madeira University, Funchal, Portugal; ^2^Research Center in Sports Sciences, Health Sciences and Human Development (CIDESD), Vila Real, Portugal

**Keywords:** handball, observational methodology, center back, attack, patterns of play

## Abstract

In the last decades, observational methodology has been widely used in scientific research in sport, namely in the study of team games like handball, due to its characteristics. Handball, in addition to being a collective, complex, dynamic, and interactive sport, has its own characteristics and demands a performance analysis that takes into account the context and interaction between different factors and variables. The present study analyzes how the different numerical relations in an attack can change the center back’s patterns of tactical behavior. Observational methodology and a mixed *ad hoc* instrument combining field format and category systems appropriately validated by experts were used. Data were taken from 20 matches involving teams classified in the first four places in the 2017 Men’s World Championship. These were recorded from TV broadcasts, and the total number of offensive sequences carried out in an organized attack game method (*n* = 990) was analyzed. In each of the sequences carried out in an organized attack, the numerical relation in attack or defense was observed and recorded. In addition to this, it was verified whether the attacking team maintained or replaced the goalkeeper by a field player in the attack. Both sequential analysis techniques with lags, prospective, and retrospective, as well as polar coordinate analysis, were used. Results have shown that there are different behavioral patterns of the center back in the three different situations of numerical relation. Another element that stood out was that in numerical equality with the defense and no goalkeeper at the goal, the center back opted for greater security and less risk of loss of the ball.

## Introduction

Team sports entail complexity, opposition, and cooperation. They also involve continuous interaction of the players’ behaviors among themselves and with the context, be it in the place of the game, the zone of the field where the actions take place, the elapsed playing time, the partial score, or the numerical relation (Garganta, 1997; Tavares, 1999; Ribeiro and Silva, 2002; Prudente, 2006, unpublished; Garganta, 2009; Schrapf et al., 2017). In these sports, behaviors are usually emergent and can derive from the individual characteristics, the possibilities that the context offers, and the characteristics of the tasks performed by the players (Travassos et al., 2010, 2013; Rodriguez and Anguera, 2014).

Handball, which is a collective sport, is defined by Balagué et al. (2013) as a dynamic and interactive as well as a complex system.

In this sport, the strategic tactical behavior is crucial (Prudente, 2006, unpublished; Sequeira, 2012; Leite and Barreira, 2014; Sousa et al., 2015; Lozano et al., 2016; Prudente et al., 2017), and the performance is the result of the interaction between different factors and variables. These are interesting aspects to research further to understand how they interact with each other. As referred by Garganta (2009), in team sports, a fundamental aspect is to understand the tactical performance of the players in order to improve their performance and effectiveness. A study that identifies the constraints that influence tactical performance and allows a deeper knowledge about the complexity of the game and its interactions is justifiable.

Handball games with different numerical relations are frequent and have increased in the last competitions (Pueo and Espina-Agullo, 2017). These game situations lead to the creation of different configurations and consequently the adaptation of behaviors of the players and teams (Prudente, 2006, unpublished). Studies about the game in different numerical relationships, or in which this variable was analyzed, have been made by different researchers (Anti, 1999; Moreira and Tavares, 2004; Prudente et al., 2004; Debanne, 2005; Prudente, 2006, unpublished; Silva, 2008, unpublished; Espina-Agullo et al., 2012; Sierra-Guzmán et al., 2015; Lozano et al., 2016; Beiztegui-Casado et al., 2017; Pueo and Espina-Agullo, 2017; Ferrari et al., 2018). Nonetheless, this is a subject that deserves a new approach.

In the last decade, handball and its specific playing positions have evolved and changed to the extent that it has become a different game in what concerns rules and speed, thus making players have a different approach to the game.

Since 2016, games and rules have been changed by the International Handball Federation (IHF). Since the new Rule 4:1 paragraph 3 and the Rules 4:4-5-6-7, players have been in an adaptation process that has led to the evolution and transformation of the various specific playing positions, including the center back.

According to Moreira and Tavares (2004) and Ohnjec et al. (2013), the main game method of attack is the organized attack. However, with the aforementioned rules and the changes they brought, we cannot help but to question how these influence this sport. We aim to verify how the behavior of the center back changes with the different numerical relations now that the new goalkeeper rule has been enforced by the IHF.

Though the center back player is still acknowledged as the playmaker, this seems to be somehow changing, hence our interest in conducting a more thorough study in this area.

## Materials and Methods

### Sample

The World Championships are crucial moments to gauge the play levels and the evolution trends. This high-level competition enables a privileged moment for the study of the players’ and teams’ behaviors, allowing innovations and applications at the tactical-technical and strategic levels to be observed, more precisely the adaptation to the new rule of the goalkeeper/field player, this way giving teams new possibilities to explore.

The sample used was the total number of offensive sequences (*n* = 990) carried out in an organized attack game method in 20 matches involving the teams classified in the first four places in the 2017 Men’s World Championship.

There were 102 attacks in numerical superiority with a goalkeeper and 19 attacks in numerical superiority without a goalkeeper (empty goal), 24 attacks in numerical inferiority with a goalkeeper and 25 attacks without a goalkeeper (empty goal), and 714 in numerical equality with a goalkeeper and 106 attacks without a goalkeeper (empty goal).

### Procedures

Observational methodology and a mixed *ad hoc* instrument consisting of a field format with category systems duly validated by seven experts (all of which are coaches and have first league experience as well as 15 years’ experience as coaches; four of them were national team coaches). The unique characteristics of observational methodology have made it the preferred method of scientific research in sport and team games, namely in handball, which have been using it over the last decades (Anguera and Hernandez-Mendo, 2014).

In each game, all the offensive sequences carried out in an organized attack were observed.

The offensive sequence is defined as from the moment the team regains possession of the ball until the moment the ball is lost. The end of this unit of observation can be caused by finalization, a committed foul, the loss of the ball due to a technical fault, or the ball leaves the field game by intervention of the opponent. The moment a sideline or corner launch is made, it is considered a new sequence.

The games were observed from recordings obtained from TV broadcasts and directly recorded on a MacBook Air 11″ computer, 2012.

In each of the sequences carried out in an organized attack, the numerical relation in the attack/defense was observed and registered. In addition to this, whether the attacking team maintained a goalkeeper (goalkeeper attack) or the goalkeeper was replaced by a field player in the attack (attack without goalkeeper – Empty Goal) was taken into account.

Exclusion criteria were established. All periods that were more than 10% unobservable (Anguera, 1990) were eliminated. All sequences where another play method other than the organized attack was used were eliminated. The play method of an organized attack is an offensive system with the participation of all attacking players in the presence of an opposition organized in a defensive system (Anti et al., 2006).

### Instrument

The following variables were studied: (1) the organization of the attack: whether it was organized or unorganized, and in the case of an organized attack whether it was done through verbal, non-verbal, or both verbal and non-verbal communication; (2) the numerical relation attack defense taking into account the equality, the superiority and the inferiority with or without the goalkeeper; (3) the tactical means used in the pre-finalization, whether it was coordinated or carried out by the center back: the position changes, the curtain, the second pivot entrance, the cross and block with or without the ball, or the absence of tactical means; (4) the finalization zone of the offensive sequence – a field map was designed with 12 zones: 11 zones on the offensive side (left/right wing, left/central/right zones from 6 to 9 m; left/central/right zones from 9 to 15 m; left/central/right zones from 15 to 20 m) and 1 zone on the defensive side; (5) the mode of finalization due to the participation of the center back in the offensive sequence taking into account the assistance, shots, shots after feint, dribble and break passes, ball losses, and no relevant action—this means that the center back is playing without influencing the offensive sequence, which is considered after the center back has passed the ball but in the next fourth pass no finalization of the sequence occurred; (6) the result of the finalization is no shots, goals, no goals, 7 m penalty with goals, or no goals; and (7) the partial score can be based on a draw, a balanced victory, an unbalanced victory (by 4 or more goals), a balanced defeat, and an unbalanced defeat (by 4 or more goals).

### Quality of Data

Data were observed and recorded using Lince 1.2.1 software analyzed with GSEQ 5.23, HOSAN 1.6.3.3.5, and SAGT 1.0 software.

Observational methodology entails collection of quality data for research. To guarantee reliable results, data were collected using the Cohen’s *κ* and the *G* tests. While in the former, intra- and inter-observer were used; in the latter, the C/O and O/C models were applied.

Observer reliability was tested after the training period (between 0.83 and 0.96 for the intra-observer and 0.79 and 0.93 for the inter-observer) and prior to the observation sessions. During the process of observation, the collection of data was checked for reliability after every four sessions and in the last session of observation.

After data collection using the Lince software, it was exported to GSEQ to be explored with simple statistics and Cohen’s *κ* test using GSEQ functions “Compute Simple Statistics” and “Compute Kappa.” The results obtained in the Cohen’s *κ* test were between 0.81 and 0.97, both in the intra- or inter-observers.

Next, SAGT software was used to make the generalizability test, which excludes the sources of the error variations caused by different observers, the observation instrument, and the categories used (Blanco et al., 2014). The results obtained in the *G* test were 0.990 for the C/O model and 0.001 for the O/C model.

### Statistics

Finally, the data were transferred to HOISAN to be analyzed by polar coordinate analysis. This analytical technique enables the reduction of the amount of information and helps build a vector map that combines the prospective and retrospective perspectives. It aids in the perception of the interrelations between the variables of the observation instrument, and it is being used much more in studies in handball and other team sports (Silva, 2008, unpublished; Rodriguez and Anguera, 2014; Sousa et al., 2015).

HOISAN applies sequential analysis techniques with lags, simultaneously prospective and retrospective (Hernandez-Mendo, 1999). Using the “Dibujar vectores,” a vector map with different conducts was made. This assisted in the analysis of not only the type of relationship between the different variables considered (regarding the position of the vectors in the quadrants related to these variables) but also the intensity of this relationship (regarding the vector length).

## Results

In this analysis, focal conduct was used when analyzing the Numerical Relation (equality, superiority, and numerical inferiority, with goalkeeper and with empty goal) and how it conditioned the different categories: the Tactical Means Pre-Finalization (change positions, curtain, second pivot entrance, cross, block with ball and block without a ball, without tactical means used); the Mode of Finalization of the center back participation in the offensive sequence (assist, shot, shot after feint, shot after dribble, break pass, ball loss, and no relevant action); the Result of Finalization (no shot, goal, no goal, 7 m goal, and 7 m no goal); and the Partial Score (draw, balanced victory, unbalanced victory, balanced defeat, and unbalanced defeat).

**Figure 1:**
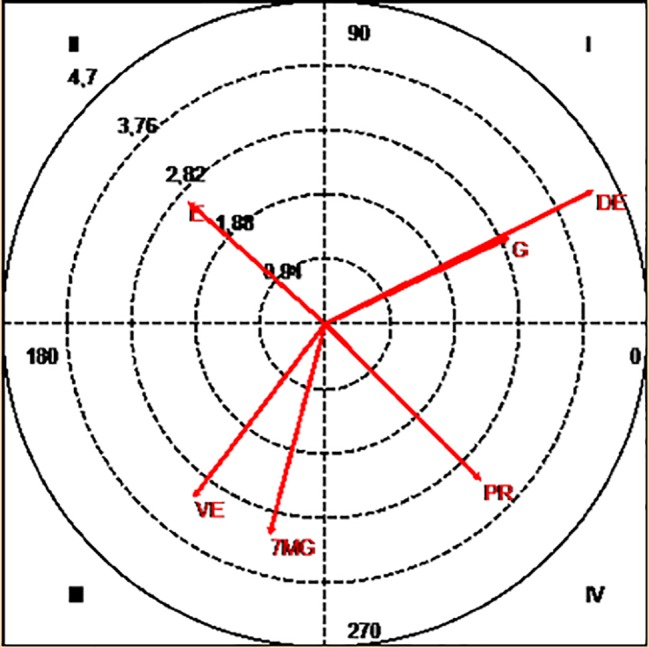
Polar coordinates map: focal conduct “equality with goalkeeper.” Conditioned conducts: 7MG, goal by 7 m; DD, unbalanced defeat; DE, balanced defeat; E, draw; G, goal; VD, unbalanced victory; VE, balanced victory; PR, break pass.

If the focal conduct is the “numerical equality with a goalkeeper” and the conditioned conducts the tactical means of pre-finalization, the mode and the result of the finalization, and the partial score, the following results were obtained as shown in Figure 1.

Results show that the mutual activation relation of the given conduct and “goal” (3.0), “balanced defeat” (4.4), and the “break pass” (3.24) is significant, as can be seen in Figure 1. Mutual inhibitory relationships were also detected with the “balanced victory” (3.2) and a probability of inhibiting the occurrence of “7 meters with a goal” (2.97).

**Figure 2:**
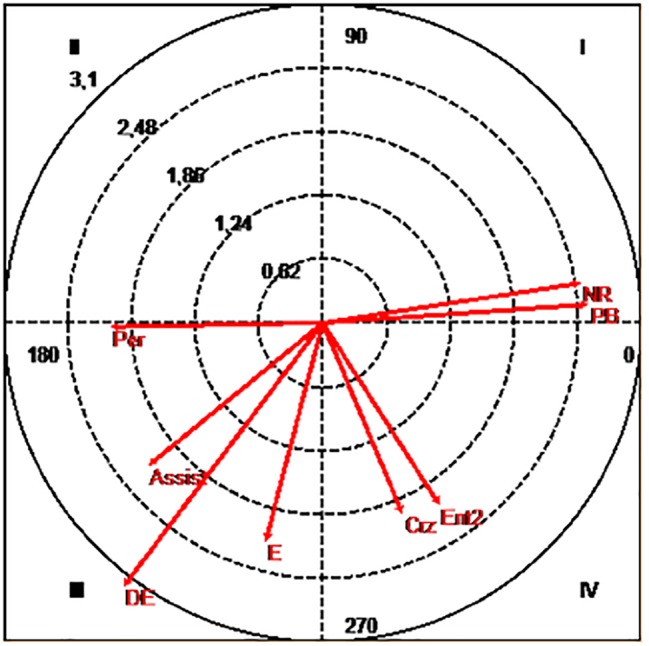
Polar coordinates map: focal conduct “equality without goalkeeper (empty goal)”. Conditioned conducts: Assist, assist; Crz, cross; DE, balanced defeat; E, draw; Ent2, second pivot entrance; NR, no shot; Per, change; PB, ball loss.

Our study also took into account the relation of numerical equality but with empty goal, as can be seen in Figure 2. In this case, the team plays without the goalkeeper, replacing him by a field player to have equality in the attack and play 6 × 6.

Significant findings were obtained from this relation regarding the mode and result of finalization, as well as pre-finalization and partial score. In this case, a significant probability of the given conduct to activate the occurrence of the “ball loss” (2.6) and the “no shot” (2.56) was verified.

Furthermore, it was also established that by relating the same conduct with the tactical means of Pre-Finalization, the probabilities of inhibition of the “second pivot entrance” (2.12), “cross” (2.03), and the “change positions occurrence” (2.08) were significant. In addition, a significant probability of a mutual inhibition with the “assist” (2.2) as well as the “balanced defeat” (3.23) and “draw situation” (2.21) was also detected.

**Figure 3:**
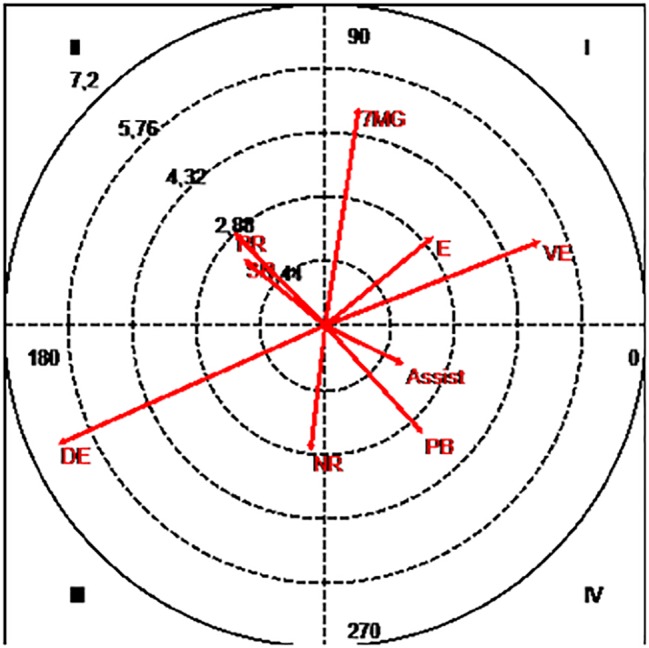
Polar coordinates map: focal conduct “superiority with goalkeeper”. Conditioned conducts: 7MG, Goal by 7 m; Assist, assist; DE, balanced defeat; E, draw; NR, no shot; PB, ball loss; PR, break pass; SR, no relevant action; VE, balanced victory.

Significant relationships were found (Figure 3) when conducting the polar coordinate analysis based on the focal and conditioned conducts. The former refers to the “numerical superiority with goalkeeper” and the latter to the Pre-Finalization, the Finalization Mode and Result, and the Partial Score.

Another significant verification made had to do with the probability of the focal conduct being mutually excitatory with the conducts “7 meters goal” (4.97), “draw” (3.15), and “balanced victory” (5.2). In addition to this, this focal conduct is also positively associated with “assist” (1.97) and “ball loss” (3.28).

It is important to know that there is a significant probability that the focal conduct activates the occurrence of the “no shot” (2.36), “break pass” (2.93), and the “no relevant action” (2.36). Equally significant is the probability of non-association with “balanced defeat” (6.56).

**Figure 4:**
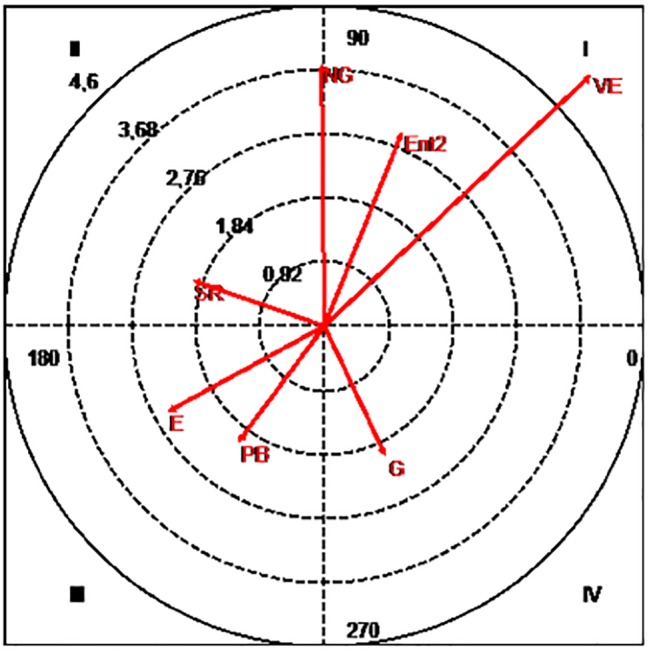
Polar coordinates map: focal conduct “Superiority without goalkeeper.” Conditioned conducts: E, draw; Ent2, second pivot entrance; G, goal; NG, no goal; PB, ball loss; SR, no relevant action; VE, balanced victory.

When the focal conduct corresponds to the “numerical superiority without goalkeeper” and the conditioned conducts refer to the tactical means of Pre-Finalization, the Mode and the Result of the Finalization, and the Partial Score, the results obtained are those shown in Figure 4.

From the results obtained, the mutual activation relation between the focal conduct and the conditioned conducts “second pivot entrance” (2.99) and “balanced victory” (5.22) is significant. They also show that this conduct is positively associated with “goal” (2.05), but there is a mutual inhibition with the “ball loss” (2.07) and “draw” (2.54). This conduct is also related to a significant probability of the inhibition of the conducts “no goal” (3.73) and “no relevant actions” (1.99).

## Discussion

The aim of this study is to analyze the behavior of the center back in the organized attack and in the different numerical relationships in which his team chooses to play with or without a goalkeeper, which is called empty goal and entails replacing him with a field player as permitted by the current rules.

Given the characteristics of the rules of the game – with their progressive penalties, and the possibility of players being punished with 2-minute exclusions, with disqualification due to accumulation of exclusions and with direct disqualification – sports confrontation in different numerical relationships is clearly a characteristic of the current handball game (Prudente, 2006, unpublished). With the current configuration of the rules, the game takes place with variations in the numerical relation between the two teams, not only in the attack but also in the defense. The most common relationships are still 6 × 6, 6 × 5, 5 × 6, and 5 × 5 with goalkeepers, but since the change of the goalkeeper rule, the 7 × 6 and 6 × 6 game situations with empty goal are becoming more common.

Significant values of activation or inhibition relationships were detected between the variables of the observation system used, namely with the Mode of the Finalization, but only when there were situations of numerical equality, i.e., with goalkeeper and with empty goal, and in numerical superiority with and without a goalkeeper. This finalization was considered only when made by the center back or by another player until four passes after the center back passes the ball, during an offensive sequence in an organized attack. Other variables considered were the Result of Finalization, made by the center back or by the player assisted by him, and the tactical means used prior to the finalization in which the center back was directly involved. The Partial Score was also used to corroborate if the result influenced the center back’s decisions.

As mentioned before, this is a new rule that came into effect in 2016. Though there is not much in the literature to enable a comparison and/or forward a discussion, our findings are significant and show that this new rule is changing the approach of the coaches regarding the game, as well as how certain players, namely the center back, are adapting to these changes.

### Numerical Equality With Goalkeeper

Numerical equality with goalkeeper and the organized attack are the most common situations and the most frequent (Anti, 1999; Debanne, 2005; Prudente, 2006, unpublished; Silva, 2008, unpublished). This study, which was based on 714 offensive sequences in a total of 990 that were played in an organized attack, also corroborates this.

From the results of the polar coordinates, it is evident that the center back’s patterns of play are associated with the probability of activation of the “goal” (2.73) and “break pass” (3.24). When interpreting the results, regarding the numerical relation with a goalkeeper, one verifies that the center back has a higher level of participation in the match and in decisive actions. Although in this situation the probability of scoring a goal is high, there is equally a significant probability of “ball loss” due to the number of actions he performs in the game. In the observation instrument, “no relevant actions” corresponds to the situations when the center back does not finalize, continues playing, or loses the ball after more than four passes. This reinforces the idea that the center back is the player who chooses group movement and coordinates his playmates; therefore, he has a key role in the perceptions of the affordances in team handball (Rodriguez and Anguera, 2018). Other results from the same analysis show that the significant probability of this numerical relation also inhibits the “7mGoal” (2.97). The significant probability of the occurrence of “break pass” is linked to the fact that the center back is the decision maker and responsible for the cooperation situations (2 vs. 2) with pivot players (Sousa et al., 2015; Rodriguez and Anguera, 2018). This focal conduct is also associated with the occurrence of “unbalanced defeat” (3.23) and “draw” (2.21) situations. This can be explained by the fact that in stress games situations, the center back is more inside the game, which is crucial in these situations.

### Numerical Equality With Empty Goal

This new situation is promoted by the changes of the rules with which the teams can choose to play with an empty goal, replacing the goalkeeper with a field player, in order to attack in numerical equality when they have a player penalized with 2 min. The number of sequences observed was 106 of a total of 990 played in organized attacks. Although this is a relatively recent issue, Beiztegui-Casado et al. (2017) have studied the goalkeeper as a field player in numerical inferiority situations, in the context of the old rules of handball, in the World Women’s Championship 2015. They verified that in 927 offensive sequences of numerical inferiority, teams chose to play with a goalkeeper as field player 154 times, meaning that 16% of the sequences were made in numerical inferiority. In this study, considering the total of sequences observed played in numerical inferiority, a total of 108 (only 95 occurred, i.e., 87%) were played with empty goal, meaning numerical equality in attack. Our study, based on a sample from the World Men’s Championship 2017, reached a low result (11.5%) but more than 10%. These results show that teams started to explore the new rules about the goalkeeper, and this change is now used by coaches and teams. From the results, it can be inferred that the focal conduct “numerical equality with empty goal” activates “no shot” (2.56), “ball loss” (2.6), “cross” (2.03), and “second pivot entrance” (3.46) and inhibits the “assist” (2.2) and the “change positions” (2.08) of the center back. A growing change in patterns regarding the previous studies conducted by Beiztegui-Casado et al. (2017) can be observed. According to them based on the results they found, they stated that the use of the goalkeeper as field player favors goal scoring and was a good option to cancel the numerical inferiority in the attack. Our findings also showed that in this situation, the center back actually promotes the use of group tactical means prior to finalization (cross and second pivot entrance). This situation can be explained by an attempt to keep the ball longer, even though the results show that they can be associated with negative aspects such as “ball loss” (2.6) and “no shot” (2.56).

It is our assumption that the differences between the results obtained by Beiztegui-Casado et al. (2017) and this study could be explained by the fact that their sample was in the Women’s Handball World Championship 2015, prior to the modification of the goalkeeper participation rules.

An association of inhibition was detected between focal conduct and partial score (“draw” 2.21 and “balanced defeat” 3.23), which can be explained by the decision to play with a goalkeeper. This occurred usually when the team was winning and felt it did not need to take risks.

### Numerical Superiority With Goalkeeper

Results show that teams used 102 sequences in “numerical superiority with the goalkeeper” and that the focal conduct Numerical Superiority with Goalkeeper activates the “7 m goal” (4.97), “assist” (1.97), and “ball loss” (3.28) but inhibits “no shot” (2.36), “break pass” (2.93), and “no relevant actions” (2.36).

We can explain the higher probability of occurrence of the “7 m goal” by the higher probability of shoot from 6 m or without opposition, thus promoting the occurrence of the 7 m penalty. This is a valid explanation for the probability of inhibition of “no shot,” “no relevant actions” realized by the center back, as well as the inhibition of the “break pass.”

When relating focal conduct with “partial score” (“balanced victory” 5.2 and draw 3.15), an association of activation of conditioned conducts was verified. This clearly shows that teams prefer to maintain the goalkeeper when they have “numerical superiority” and are winning.

### Numerical Superiority Without Goalkeeper

Results show that teams have only used 19 sequences in numerical superiority when they chose to play with the empty goal. These situations (7 × 6 in attack) lead to some positive and significant results, even though it was the smaller sample of our study. Teams that play with this organization are positively successful in their ventures. In other words, this is associated with “goal” (2.05) and inhibits the situations where there are “ball loss” (2.07) and “no goal” (3.73). Our results show that when playing with seven field players, there are usually two pivots, that is why there is a connection with the “second pivot entrance” (2.99). In a 7 × 6 configuration, there a significant association with “balanced victory” (5.22). In our opinion, these results show that this situation is becoming much more common and there are more teams exploring it.

These results show that the center back’s playing patterns are different depending on the configuration of play related to the numerical relation. Unlike Rodriguez and Anguera (2018), who studied whether these patterns were differentiated by the player that occupied that playing position, our main focus is on whether or not the new goalkeeper rules have influenced the center back’s actions.

This study is only a first approach that renders some knowledge and information that can be shared with coaches so that they can explore the possibilities that this new rule offers when letting a field player replaces the goalkeeper. By knowing more about the different patterns of play of the center back, solutions can be sought and found for the tactical training of this player. This way, players and coaches will have a better perception of the possibilities offered by the contextual variable numerical relation.

Different patterns of behavior of the center back in different numerical relationships were identified and analyzed, but it is our recommendation that in the future, studies should be continued and take into account context variables such as defensive systems and playing time. This will provide a deeper understanding of the center back’s actions and tactical relations during organized attacks.

Fully aware that evolution in the game trends is to be expected, we advise these studies to be extended and larger samples be included so as to consistently analyze the participation of the center back in situations of numerical superiority with empty goal (7 × 6).

## Conclusions

After the analysis and discussion of the results, it can be concluded that the observed numerical relations, whether of equality or superiority, influenced the behavior and actions of the center back during the game and in the team dynamic. There is no doubt that the center back’s playing patterns are different depending on configuration of play related to the numerical relation.

## Data Availability Statement

The datasets generated for this study are available on request to the corresponding author.

## Author Contributions

JP, DS, AR, and AC drafted the work and provided approval for the version to be published.

### Conflict of Interest

The authors declare that the research was conducted in the absence of any commercial or financial relationships that could be construed as a potential conflict of interest.
